# 

**Published:** 1899-03

**Authors:** 


### PERSONALS.

Dr. Cooper Curtice, formerly of Moravia, N. Y., has already joined with his colleagues in North Carolina for the attainment of better veterinary sanitary regulations for the people of that State.

Dr. W. E. Wight, of Delaware, Ohio, is taking a post-graduate course of instruction at the McKillip Veterinary College, Chicago.

Drs. Lloyd Zaner, of Stillwater, Pa., and F. H. Mackie, of Fair Hill, Md., were visitors to Philadelphia in January.

Dr. John Faust, of Poughkeepsie, N. Y., has been seriously ill with the "grip," complicated with bronchitis. He has recently received an appointment as one of the Health Commissioners of that city.

Dr. G. E. Griffin, of the Fifth U. S. Cavalry, was a visitor to New York City in January.

Dr. Robert Ellis, Secretary of the New York County Veterinary Medical Association, was on the sick-list in January.

Dr. Leonard Pearson in January addressed the Bibliographical Club of Philadelphia on "Veterinary Science."

Dr. J. C. McNeil, of Pittsburg, was a visitor to Harrisburg at the inauguration of Governor Stone, and subsequently continued his trip to Philadelphia.

Dr. Harry Walters, of Wilkesbarre, is the promoter of large commercial interests in connection with which he is a frequent visitor to Greater New York and Philadelphia.

Dr. William G. Shaw, who since last June has been connected with the Pennsylvania Livestock Sanitary Board, has received an appointment to a position in the Bureau of Animal Industry, and been assigned for duty at Cincinnati.

Drs. S. L. Bearer, of Kirkman, Iowa, in connection with his practice, conducts a large wholesale drug business.

Dr. H. M. Hunter, of Tulare City, California, holds the official position of veterinarian for Tulare County of the Golden State.

Dr. Leonard Pearson spent several days in Harrisburg during the inaugural ceremonies of Governor Stone.

Dr. T. Earle Budd, formerly of Woodbury, N. J., has become a resident practitioner of Orange, in the same State.

Dr. M. O'Connell has returned from his Pacific coast sojourn in very much improved health. He has received again the appointment of City Veterinarian for the municipality of Holyoke, and also an appointment at the hands of Governor Wolcott, of Massachusetts, on the Board of Cattle Commissioners—a position which he has filled for a number of years with credit to the profession.

Professor John Uri Lloyd, the author of *Etidorpha*, will contribute to the April number of *The Coming Age*, an article on "Do Physicians and Pharmacists Live on the Misfortunes of Humanity?" Surely, the subject is a topic of peculiar interest.

Dr. G. E. Nesom fills the rôle of veterinarian to the South Carolina Experiment Station and instructor in veterinary science at Clemson College of Agricultural and Mechanic Arts.

Dr. Simon Beattie, of Madison, Wisconsin, conducts a veterinary infirmary in conjunction with his practice.

Dr. J. Stewart Lacock, of Allegheny, officiated as veterinarian to the Pittsburg pet-dog show in March.

Dr. W. H. Dalrmyple, of Baton Rouge, was active in making the meeting of the Stockbreeders' Association of that State, in January, a thorough success.

The following veterinarians in the army have been ordered abroad: Drs. G. E. Griffin, fifth cavalry, Porto Rico; R. G. Corcoran and M. J. Treacy, eighth cavalry, Nuevitas, Cuba; C. D. McMurdo and Daniel Lemay, seventh cavalry, Havana, Cuba; W. V. Lusk, second cavalry, Cuba.

Drs. D. E. Salmon and A. S. Alexander contributed articles to the February number of the *Breeders' Gazette.*

Dr. W. L. Williams, of Ithaca, N. Y., was a visitor to the veterinary colleges at Philadelphia and New York the latter part of February.

Dr. Guildin R. Hartman has again resumed an interest in the canine and feline infirmary at 914–916 Diamond Street, Philadelphia, Pa.

